# Involvement of Phosphatidylserine and Triacylglycerol in the Response of Sweet Potato Leaves to Salt Stress

**DOI:** 10.3389/fpls.2019.01086

**Published:** 2019-09-10

**Authors:** Yicheng Yu, Meng Kou, Zhonghui Gao, Yang Liu, Ying Xuan, Yaju Liu, Zhonghou Tang, Qinghe Cao, Zongyun Li, Jian Sun

**Affiliations:** ^1^Jiangsu Key Laboratory of Phylogenomics and Comparative Genomics, School of Life Sciences, Jiangsu Normal University, Xuzhou, China; ^2^Key Laboratory for Biology and Genetic Breeding of Sweet Potato, Sweet Potato Research Institute (CAAS), Xuzhou, China

**Keywords:** salt stress, sweet potato, lipidomics, phosphatidylserine, triacylglycerol, K^+^/Na^+^ homeostasis, PM H^+^-ATPase

## Abstract

Lipid remodeling plays an important role in the adaptation of plants to environmental factors, but the mechanism by which lipid remodeling mediates salt stress response remains unclear. In this study, we compared the root and leaf lipidome profiles of salt-tolerant and salt-sensitive sweet potato cultivars (Xu 22 and Xu 32, respectively) under salinity stress. After salt treatment, the leaf lipidome showed more significant remodeling than the root lipidome in both cultivars. Compared with Xu 32 leaves, Xu 22 leaves generally maintained higher abundance of phospholipids, glycolipids, sphingolipids, sterol derivatives, and diacylglycerol under salinity conditions. Interestingly, salinity stress significantly increased phosphatidylserine (PS) abundance in Xu 22 leaves by predominantly triggering the increase of PS (20:5/22:6). Furthermore, Xu 32 leaves accumulated higher triacylglycerol (TG) level than Xu 22 leaves under salinity conditions. The exogenous application of PS delayed salt-induced leaf senescence in Xu 32 by reducing salt-induced K^+^ efflux and upregulating plasma membrane H^+^-ATPase activity. However, the inhibition of TG mobilization in salinized-Xu 22 leaves disturbed energy and K^+^/Na^+^ homeostasis, as well as plasma membrane H^+^-ATPase activity. These results demonstrate alterations in the leaf lipidome of sweet potato under salinity condition, underscoring the importance of PS and TG in mediating salt-defensive responses in sweet potato leaves.

## Introduction

Soil salinization is a major problem in agriculture worldwide. Salt stress caused by excess sodium chloride (NaCl) in soil disturbs many plant physiological processes (Munns and Tester, 2008). Plants have developed several mechanisms, such as remodeling membrane transporter activity, which includes the regulation of plasma membrane (PM) H^+^-ATPase activity, to prevent the damage caused by salt stress (Munns and Tester, 2008; Türkan and Demiral, 2009; Shabala and Pottosin, 2014). PM H^+^-ATPase contributes to the pumping of Na^+^ from the cytosol to the external medium by driving PM Na^+^/H^+^ antiport activity and to limiting K^+^ efflux from the cytosol by blocking the depolarization-activated outward K^+^ channel (Tester and Davenport 2003; Chen et al., 2007; Sun et al., 2009a; Sun et al., 2009b; Chen et al., 2014). Thus, PM H^+^-ATPase is an essential component in the mediation of cellular K^+^/Na^+^ homeostasis and a crucial factor in the survival of plants under salinity stress (Shabala and Cuin, 2008).

Plant salt tolerance is associated with the widespread rewiring of cellular metabolic processes, including the remodeling of lipid metabolism (Darwish et al., 2009; Krasensky and Jonak, 2012; Mansour et al., 2015; Natera et al., 2016; Yu et al., 2018). Lipids are the fundamental components of biological membranes and play essential roles in trafficking, signal transduction, and sorting of macromolecules and are particularly important in the adaptation of plants to environmental stresses (Okazaki and Saito, 2014). Lipid affects membrane integrity, permeability, fluidity, and transport protein activity under salinity condition (Mansour et al., 2015). Linoleic acid (C18:2) stimulates PM H^+^-ATPase activity and maintains K^+^/Na^+^ homeostasis in barley roots (Yu et al., 1999). Salt stress-triggered production of oleic acid (C18:1), linoleic acid (C18:2), and linolenic acid (C18:3) contributes to the activation of PM H^+^-ATPase activity in *Arabidopsis* (Han et al., 2017). These results suggest that polyunsaturated fatty acids play an essential role in mediating plant salt tolerance. In agreement with this view, Zhang et al. (2012a) reported that fatty acid desturase FAD2 is required for salt tolerance in *Arabidopsis*. Phosphatidic acid (PA), which is a biologically active lipid molecule, plays an important signaling role in mediating downstream salt defensive responses (Testerink and Munnik, 2005; Hong et al., 2010; Hou et al., 2016). This result is supported by the evidence that the phospholipase Dα1 (PLDα1)-deficient mutants of *Arabidopsis* are much more sensitive to salt stress than the wild type (Yu et al., 2010). In addition, PLDα1-derived PA promotes microtubule stabilization and salt tolerance in *Arabidopsis* (Zhang et al., 2012b). Structure phospholipids, including phosphatidylcholine (PC), phosphatidylethanolamine (PE), phosphatidylinositol (PI), and phosphatidylglycerol (PG), play different roles in plants’ response to salinity stress (Mansour et al., 2015). The increased PM abundance of PI is correlated with salt sensitivity, whereas elevated PC or PG may be associated with salt adaptation (Mansour et al., 2015). Inositol (1, 4, 5) trisphosphate, which is a derivative of the PI signaling pathway, contributes to prolonged cytosolic calcium signaling and ion homeostasis regulation in salinized *Populus euphratica* callus cells (Zhang et al., 2015). Sterols, which are important structural components of cell membranes ubiquitously present in plant cells, stimulate PM H^+^-ATPase activity in corn roots. Thus, this major class of lipids is thought to be involved in the regulation of ion transport systems, which function in ion homeostasis under salinity condition (Mansour et al., 2015).

Lipidomic analysis based on electrospray ionization mass spectrometry (ESI-MS) was first reported in 1994 (Han and Gross, 1994). Since then, a large number of lipidomic analysis methods have been developed, and many biologically important lipids have been revealed in animals, plants, and microorganisms. In higher plants, lipidomics has been used for profiling the responses of membrane lipid species to different environmental factors. For example, Degenkolbe et al. (2012) profiled the alterations in a lipidome during cold acclimation in the natural accessions of *Arabidopsis thaliana* and revealed that the relative abundance of several lipid species is highly correlated with freezing tolerance. A comparative profiling of membrane lipids in drought-stressed *Thellungiella salsuginea* and *Arabidopsis thaliana* showed that increased abundance of plastid lipids (digalactosyldiglyceride) and double bond index improves the fluidity of membranes and thus increases the water stress tolerance of *T. salsuginea* relative to that of *A. thaliana* (Yu and Li, 2014). Other studies using lipidomics methods showed that the increased synthesis rates of storage lipids, such as triacylglycerol (TG), is an essential adaptive response of plant cells to high temperature stress (Mueller et al., 2015; Narayanan et al., 2016a; Narayanan et al., 2016b). Furthermore, lipidomic analysis of the mesophyll cell and bundle sheath cell chloroplasts in maize (*Zea mays*) revealed that low monogalactosyldiacylglycerol (MGDG) content in bundle sheath cell chloroplasts renders them more tolerant to salinity stress than mesophyll cell chloroplasts (Omoto et al., 2016). Although lipidomics analyses on plant response to different environment stresses have been performed, few have specifically targeted salinity stress (Natera et al., 2016; Omoto et al., 2016). Comprehensively profiling lipid composition under salinity condition in plant species with contrasting salt tolerance will shed new light on the potential functions of individual lipid species or lipid classes in salinity tolerance (Natera et al., 2016).

Sweet potato (*Ipomoea batatas* L.) is an important food and industrial crop that ranks seventh in terms of worldwide staple food production (Bovell-Benjamin, 2007). This root crop is cultivated through vegetative propagation, and the physiological and molecular mechanisms underlying intraspecific difference in salt tolerance are largely unknown. In our previous study, we investigated salt response in two sweet potato varieties at the whole plant level. Our results showed that the salt-tolerant sweet potato cultivar has a high capacity for root ion homeostasis mediation and nitrogen uptake and assimilation under salinity condition (Yu et al., 2016). In the present study, we detected leaf and root lipidome profiles of two sweet potato cultivars under salinity conditions. We discovered the important role of phosphatidylserine (PS) and TG in mediating PM H^+^-ATPase activity and salt defensive responses in sweet potato leaves.

## Materials and Methods

### Plant Materials and Treatments

The salt-tolerant variety Xu 22 and salt-sensitive cultivar Xu 32 were used in this study (Yu et al., 2016; Yu et al., 2018). Sweet potato shoots with five or six leaves were hydroponically cultured in half-strength Hoagland solution at 28°C with a photoperiod of 16 h (300 μmol m^−2^ s^−1^) for 2 weeks. Uniform rooting seedlings were subsequently cultivated in half-strength Hoagland solution with 200-mM NaCl for 7 days. Then, the tender roots and functional leaves were excised, immediately frozen in liquid nitrogen, and subsequently stored at −80°C for further lipidomics analysis.

For exogenous PS experiments, functional leaves were collected from Xu 32, pretreated with 0.1-, 0.5-, and 1.0-μM PS emulsion (Sigma, from soybean) for 24 h, then placed in a transparent culture dish containing essential nutritional elements (Knop’s solution) and corresponding concentrations of PS. NaCl (200 mM) as salt treatment was added to the culture solution. The solutions were refreshed every day, and the culture dishes were placed at 28°C with a photoperiod of 16 h (300 μmol m^−2^ s^−1^) for 6 days. Afterward, the detached leaves were collected for various physiological analyses.

For diphenyl methylphosphonate (DMP) experiments, functional leaves were collected from Xu 22 and pretreated with 25-μM DMP (Sigma) for 24 h, and salt treatment was applied as described earlier. Finally, the detached leaves were collected for various physiological analyses.

### Lipid Extraction and Lipidomics Analysis

Fresh leaf and root samples (0.1 g) were ground in liquid nitrogen to fine powder and extracted with 1.4 ml of 100% isopropanol. The mixture was transferred into 2-ml centrifuge tubes for 10-s vortex oscillation and 10-min ultrasonic treatment. The mixture was frozen at –20°C for 1 h and oscillated at room temperature. The samples were then centrifuged at 10,000×*g* for 20 min at 4°C. The supernatant was filtered with a 0.22-μM filter and transferred to a glass vial for ultra-performance liquid chromatography (UPLC)/ESI-quadrupole-time-of-flight (Q-TOF)-MS analysis. Each sample (2 µl) was injected onto a reverse-phase CSH C18 column (1.7 µm, 1 × 50 mm) by using an Acquity *I*-class UPLC system (Waters Corporation, USA). The column oven temperature was set at 55°C. The mobile phase comprised ACN/H_2_O (60%/40%) containing 0.1% formic acid and 10-mM ammonium formate (solvent A) and IPA/ACN containing 0.1% formic acid and 10-mM ammonium formate (90%/10%; solvent B). Each sample was resolved for 20 min at a flow rate of 0.4 ml/min. The UPLC gradient started with 40% B and then ramped to 43% B from 0 to 2 min, 50% B from 2 to 2.1 min, 54% B from 2.1 to 12 min, 70% B from 12 to 12.1 min, 99% B from 12.1 to 18 min, and 40% B from 18 to 18.1 min and then held for 2 min (Yu et al., 2018).

MS was performed on a Q-TOF instrument (Xevo G2-S QTOF, Waters Corporation, USA) operated in either negative (ESI−) or positive (ESI+) electrospray ionization mode with a capillary voltage of 3 kV and sampling cone voltage of 25 V in both modes. The desolvation gas flow was set to 800 l/h, and the temperature was set to 500°C. The source temperature was set to 120°C. Accurate mass was maintained by introducing a lock-spray interface of leucine–enkephalin (556.2771 [M+H]+ or 554.2615 [M−H]−). Data were acquired in continuum MSE mode from 50 to 1,500 m/z. The pooled quality control (QC) samples (generated by taking an equal aliquot of all samples included in the experiment) were ran at the beginning of the sample queue for column conditioning and every 10 injections thereafter as described previously (Yu et al., 2018). A test mix of standard metabolites was ran at the beginning and end of the process for the evaluation of instrument performance with respect to sensitivity and mass accuracy. The overlay of the total ion chromatograms of the QC samples showed excellent retention time reproducibility. The sample queue was randomized for the removal of bias. Variance was stabilized by normalizing the m/z features of the metabolites through log transformation, and the uniform empirical distribution of intensities across the samples was obtained through quantile normalization. The metabolites were selected through a receiver operating characteristic-regularized learning technique based on the LASSO penalty as implemented with R package “glmnet,” which uses a cyclical coordinate descent in a pathwise manner.

### Measurement of K^+^ and H^+^ Fluxes in Mesophyll Cells

Salt shock-triggered transient K^+^ and H^+^ fluxes were determined noninvasively with vibrating ion-selective microelectrodes (noninvasive microtest system, NMT-100-SIM-YG, Younger USA LLC, Amherst, MA, USA) as previously described (Sun et al., 2009a; Sun et al., 2009b; Yu et al., 2016; Yu et al., 2018; Liu et al., 2019). The abaxial surface of the PS-treated (pretreatment of 0.1-, 0.5-, and 1.0-μM PS for 24 h) or PS-non-treated leaves was peeled off with fine forceps to reveal the mesophyll tissue. Peeled leaves were cut into small segments and then placed on a measuring solution (0.5-mM KCl, 0.1-mM CaCl_2_, and 0.1-mM MgCl_2_, pH 5.7) in appropriate Petri dishes overnight prior to measurement for the leaves to recover from wounding response. In PS-treated leaf segments, 0.1-, 0.5-, or 1.0-μM PS was added to the measuring solution for maintenance of PS effects. In the following morning, the segments were immobilized on a new chamber containing a fresh measuring solution (without PS) for 30 min before measurement. Afterward, the steady fluxes of H^+^ and K^+^ were recorded for 10 min in the mesophyll tissue. Salt shock (200-mM NaCl) was then activated by adding NaCl stock (400 mM, pH 5.7, prepared with measurement solution), and the transient K^+^ and H^+^ fluxes were monitored for another 40 min. Net fluxes were calculated with JCal 1.0 (a free MS Excel spreadsheet; http://youngerusa.com/jcal or http://ifluxes.com/jcal).

### Plasma Membrane Vesicle Purification and Plasma Membrane H^+^-ATPase Activity Determination

An adequate quantity of leaf samples (approximately 20 g) was collected and homogenized in 20 mL of homogenization buffer containing 250-mM sucrose, 10% (w/v) glycerol, 0.5% (w/v) polyvinylpyrrolidone, 3-mM ethylenediaminetetraacetic acid, 1-mM dithiothreitol, 1-mM phenylmethylsulfonyl fluoride, 15-mM mercaptoethanol, and 25-mM Tris/2-ethanesulfonic acid (pH 7.6). The homogenate was filtered through two layers of cotton gauze and centrifuged at 13,000×*g* for 20 min. The supernatant was re-centrifuged at 80,000×*g* for 30 min. The obtained microsomal membranes were re-suspended in a buffer containing 1-mM dithiothreitol, 1-mM phenylmethylsulfonyl fluoride, and 5-mM Tris/2-ethanesulfonic acid (pH 6.5). The microsomal membranes were used in the assessment of 9-amino-6-chloro-2-methoxyacridine (ACMA) quenching and analysis of H^+^ pumping activity (Shabala et al., 2016; Yu et al., 2018). The membranes were also used in the analysis of ATP hydrolysis activity on the basis of the measured amount of released inorganic phosphorus (Liu et al., 2014; Yu et al., 2018).

### RNA Extraction and Quantitative Real-Time Polymerase Chain Reaction

Trizol reagent (Takara Bio Inc., Japan) was used to isolate total RNA from the leaf tissues. Afterward, 2 µg of total RNA was reverse-transcribed with a First complementary DNA (cDNA) kit (Takara Bio Inc., Japan). The synthesized cDNA (1 µl) was used as a template for RT-PCR amplification. The PCR products were sequenced and validated. The following primers designed to target genes were established based on our transcriptome sequences: *PHA1* (PM H^+^-ATPase; forward primer 5′- GCACACCTTGTTGACTCTACTA-3′ and reverse primer 5′-TGAGAGGACAGTTGGCATTG-3′), *DGAT1* (acylCoA:diacylglycerol acyltransferase; forward primer 5′-TGCCTGTTCATAAGTGGATGGT-3′ and reverse primer 5′-ACATAATTCCAATAAATGCCCAGA-3′), *PDAT1* (phospholipid: diacylglycerol acyltransferase; forward primer 5′- AGTCGATACTGAGGCGGAGAAAGG-3′ and reverse primer 5′CATGAACAACAGCACCCACCAAAT-3′) and *SDP1 (Sugar-Dependent 1,* TG lipase; forward primer 5′-AGGGCGATGTGACAGTTGTGATG-3′ and reverse primer 5′TCGACGCATGTGGTTGAGTATTG-3′). The resulting amplicons were between 100 and 300 bp long. The qPCR mixtures (20 µl of total volume) contained 10 µl of UltraSYBR Mixture (Beijing CoWin Biotech, China), 1.0 µl of cDNA template (10 ng), 0.5 µl of each forward and reverse primers (0.25 µM), and 7.5 µl of RNase-free water. PCR analysis was performed using an ABI Stepone plus PCR system (ABI Co., Ltd., USA). Melting curves were analyzed immediately for the confirmation of product specificity. The mean Ct value of each gene was obtained from three independent PCR experiments. The relative expression level of each target gene was normalized to that of *IbUBI* (GenBank accession number: JX177358.1), a stable internal reference gene of *Ipomoea batatas* (Park et al., 2012). This gene was amplified by using the following primers: forward primer 5′-TCGACAATGTGAAGGCAAAG-3′ and reverse primer 5′CTTGATCTTCTTCGGCTTGG-3′. Relative expression levels were calculated with the 2^–ΔΔCt^ method.

### Chlorophyll, Malondialdehyde, and Electrolyte Leakage

Chlorophyll content, electrolyte leakage, and malondialdehyde contents were determined in accordance with the method reported previously (Yu et al., 2016).

### Superoxide Anion and Hydrogen Peroxide

To determine the O_2_^.–^ production rate, 0.5 g of leaf tissue was ground to a fine powder in liquid nitrogen and then homogenized in 5 ml of extraction buffer (pH 7.8) containing 50-mM sodium phosphate, 5-mM ethylenediaminetetraacetic acid, and 1% (w/v) polyvinylpyrrolidone. After centrifugation at 12,000×*g* (4°C) for 20 min, the supernatants were collected, and the O_2_^.–^ production rate was assayed through the oxidation of hydroxylamine at 530 nm as described previously (Yu et al., 2016). For H_2_O_2_ content, root and leaf tissues (0.5 g) were ground in liquid nitrogen and then homogenized in 5 ml of cold acetone. After centrifugation at 3,000×*g* (4°C) for 10 min, the supernatants were used for H_2_O_2_ content assay. H_2_O_2_ content was assayed by analyzing the production of titanium–hydroperoxide complex at 410 nm (Patterson et al., 1984). Soluble protein concentration was determined with the Bradford assay (Bradford, 1976), and bovine serum albumin was used as the standard.

### Determination of Na^+^ and K^+^ Contents

Leaf samples were rinsed with deionized water and dried at 70°C to a constant weight. After the samples were pulverized, 0.2 g of powdered dry samples was digested in a H_2_SO_4_–H_2_O_2_ solution. The K^+^ and Na^+^ contents of the extract were determined with an atomic absorption spectrophotometer (Shimadzu AA-680, Shimadzu Ltd., Kyoto, Japan) as previously described (Yu et al., 2016; Yu et al., 2018).

### Relative ATP Content

Leaves were ground to fine powder in liquid nitrogen. Subsequently, 50 mg of powder was homogenized with 500 µl of 0.1-M hydrochloric acid for 5 min. The homogenate was centrifuged at 18,000×*g* for 10 min, and the supernatant was centrifuged again at 14,000×*g* for 20 min. ATP content was determined with the Enlighten ATP Assay System Bioluminescence Kit (Promega Corp., Madison, WI, USA). Relative ATP level was expressed as normalized luminescence.

### Statistical Analysis

Data were subjected to analysis of variance. Significant differences between means were determined by Duncan’s multiple range test. Unless otherwise stated, differences at *P* < 0.05 were considered significant.

## Results

### Salt Stress Alters Lipid Composition in Sweet Potato Leaves and Roots

Lipidomic analysis detected 524 and 330 lipid molecular species in the leaves and roots, respectively. These species belong to 15 major lipid classes, namely, the glycolipid MGDG; phospholipids PG, PC, PE, PI, PS, and PA; lysophospholipids lysophosphatidic acid (LPA), lysophosphatidylcholine (LPC), lysophosphatidylethanolamine (LPE), and lysophosphatidylserine (LPS); the storage lipid TG; the lipid intermediate diacylglycerol (DG); and the sterol derivatives (sterols) and the sphingolipids (**Supplementary Tables S1** and **S2**). PCA of the lipids identified in the leaves showed a clear separation between the varieties or between the treatments (**Supplementary Figure S1**). These results indicate a substantial difference in leaf lipidome between the varieties under control condition and suggest the substantial modulation of lipidome under salt stress.

**Figure 1:**
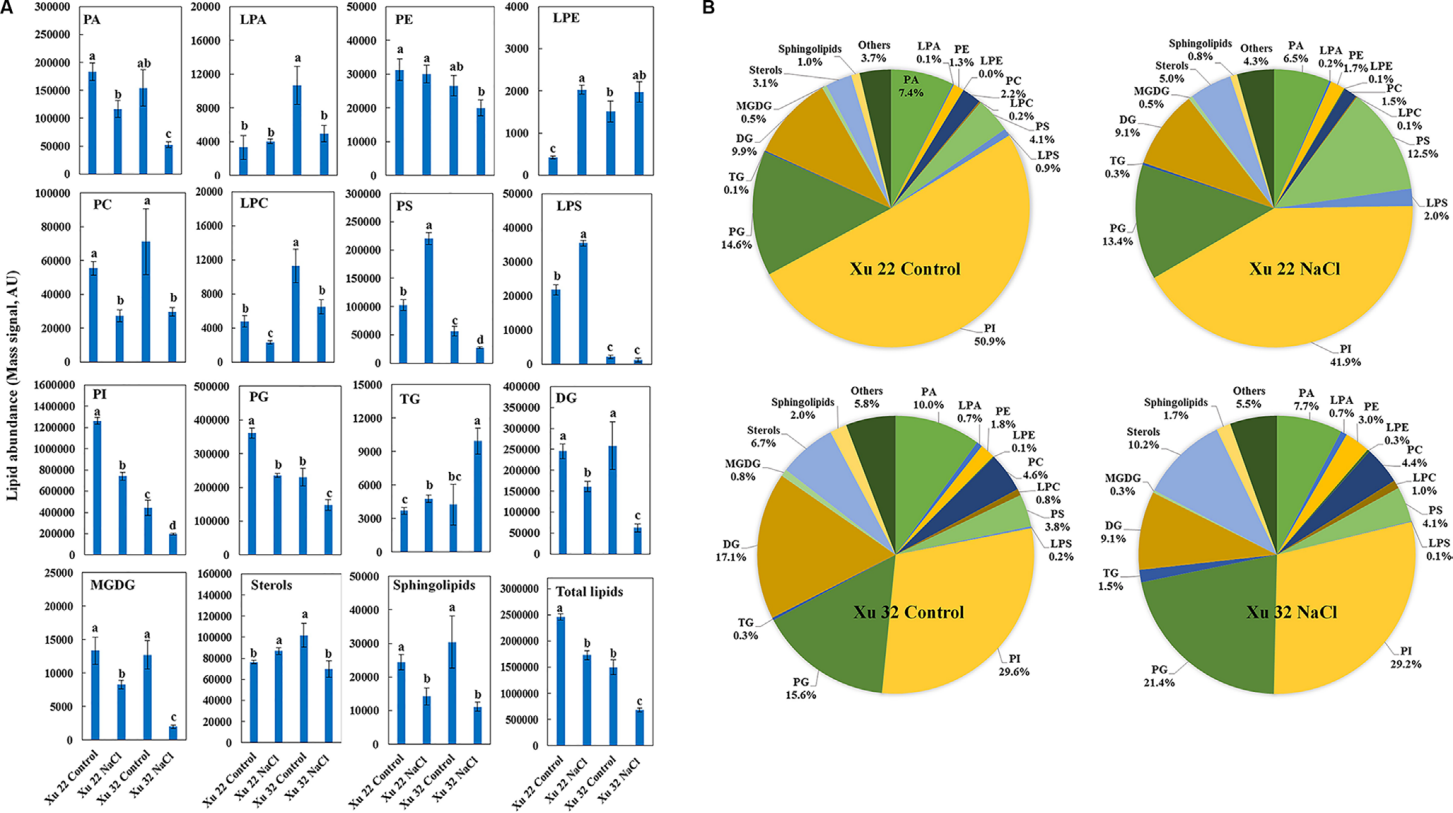
Effects of NaCl stress (200 mM for 7 days) on the total amount of lipids in various head group classes of leaves in sweet potato cultivars Xu 22 and Xu 32. **(A)** Columns represent the means of four replicates per treatment, and bars represent the standard error of the mean. Columns labeled with different letters (a–d) indicate significant difference at *P* < 0.05 (analysis of variance). **(B)** Relative abundance of lipids assigned with the major lipid classes in control and stressed samples at 7-day post-treatment of NaCl. Each lipid class is expressed as a percentage of the total amount of lipids detected. PA, phosphatidic acid; LPA, lysophosphatidic acid; PE, phosphatidylethanolamine; LPE, lysophosphatidylethanolamine; PC, phosphatidylcholine; LPC, lysophosphatidylcholine; PS, phosphatidylserine; LPS, lysophosphatidylserine; PI, phosphatidylinositol; PG, phosphatidylglycerol; TG, triacylglycerols; DG, diacylglycerols; MGDG, monogalactosyldiacylglycerol.

The amount of total lipids was significantly higher in Xu 22 leaves than that in Xu 32 leaves under the control condition mainly because of the higher amount of PI and PG detected in Xu 22 (**Figure 1A**). Major lipid classes in leaves responded in five ways to salt stress: (1) The total amounts of PA, PC, LPC, PI, PG, DG, MGDG, and sphingolipids significantly decreased in both varieties. However, the Xu 22 leaves exhibited less salt-decreased amounts of PA, DG, and MGDG than the Xu 32 leaves (**Figure 1A**). Although the salt-induced reduction in the amounts of PI and PG were more pronounced in Xu 22, the PI and PG abundance in Xu 22 were still higher under salinity condition (**Figure 1A**). (2) The total amount of TG significantly increased in both varieties after salt stress treatment. Interestingly, Xu 32 accumulated more TG than Xu 22 under salinity condition (**Figure 1A**). The percentage of TG in total lipids increased from 0.3% (control) to 1.5% (salt stress) in Xu 32 and from 0.1% (control) to 0.3% (salt stress) in Xu 22 (**Figure 1B**). (3) Salt stress markedly enhanced the total amounts of PS, LPS, LPE, and sterol derivatives in Xu 22 but reduced the abundance of these lipids in Xu 32 (**Figure 1A**). Strikingly, the percentage of PS in total lipids increased from 4.1% of the control to 12.5% in the salinized Xu 22 leaves. (4) PE abundance was unaffected by salt stress (**Figure 1A**). (5) The total amount of LPA markedly decreased in Xu 32 but was unaffected by salt in Xu 22 (**Figure 1A**).

Although the clear separation of identified lipids was not observed in the roots (**Supplementary Figure S1**), the five responses of major lipid classes to salt stress were still observed in the roots. (1) The total amounts of PA, LPE, PS, LPS, DG, MGDG, and sphingolipids were not altered by salt stress (**Supplementary Figure S2**). (2) The total amounts of PC, PE, LPA, and LPC significantly decreased in Xu 32 but were unaffected by salt in Xu 22 (**Supplementary Figure S2**). (3) The total amounts of PI and PG were markedly decreased by salt stress in both varieties, and no difference in these amounts was observed between the varieties (**Supplementary Figure S2**). (4) TG abundance was significantly enhanced by salt in Xu 32 roots but not in Xu 22 (**Supplementary Figure S2**). (5) The total amount of sterols was significantly enhanced in both varieties, and no difference in this amount was observed between the two varieties (**Supplementary Figure S2**).

**Figure 2:**
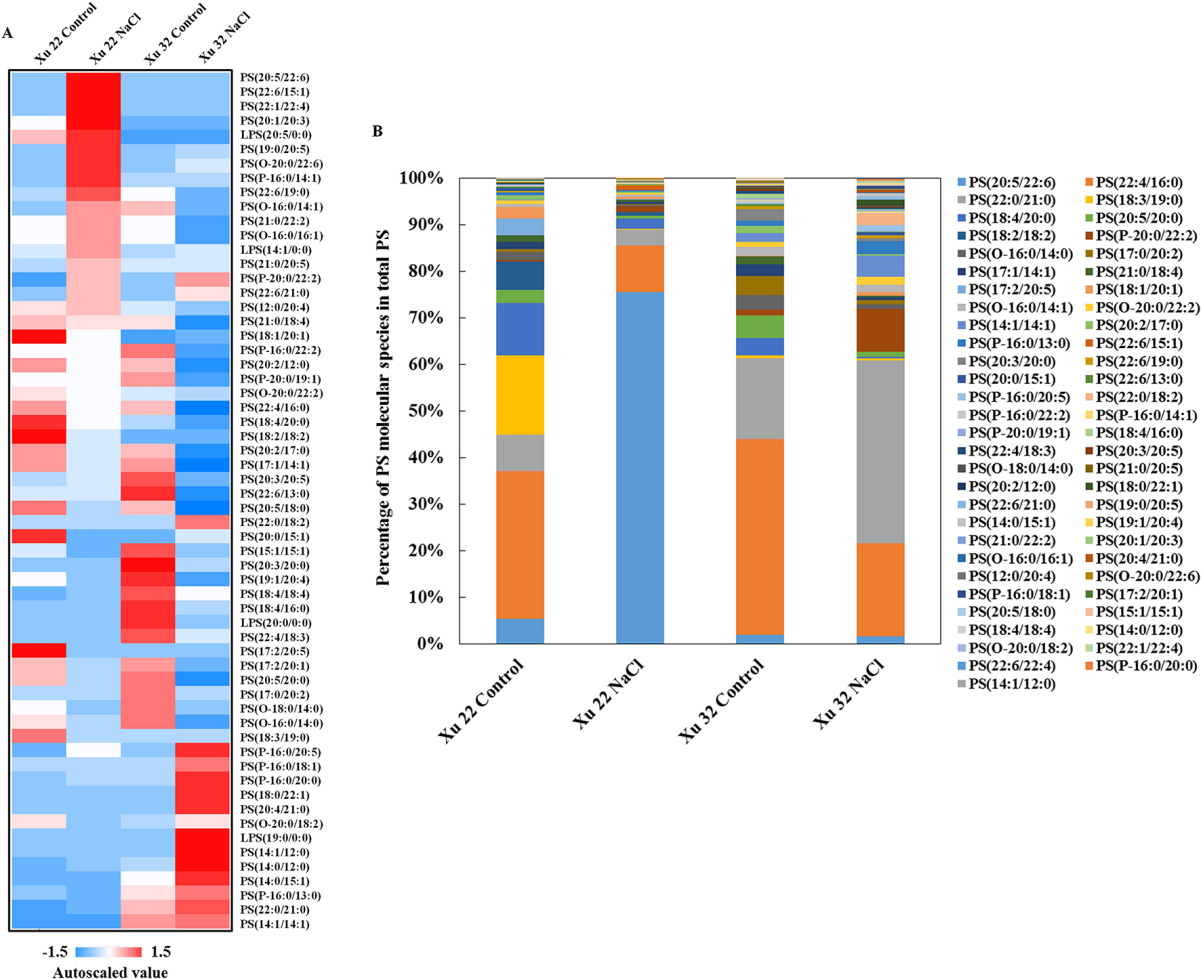
**(A)** Heat map of average autoscaled data of PS molecular species in the leaves of sweet potato cultivars (Xu 22 and Xu 32) under control and NaCl treatment conditions. Autoscaling allows for easy comparison of lipid levels in different samples. The autoscaled value of a lipid in samples is calculated as follows: [(amount of lipid in a sample) – (average amount of the lipid among all samples)]/(standard deviation of the lipid amount among all samples). **(B)** Relative abundance of PS molecular species. Each PS molecular species is expressed as a percentage of the total amount of PS detected.

We further analyzed the change trend of detected lipid species by autoscaling the data to easily compare lipid levels in different samples (**Supplementary Figure S3**; **Tables S3** and **S4**; Narayanan et al., 2016b). We focused on three major lipid classes (e.g., PS, including LPS, TG, and sterol derivatives in leaves) that showed significantly different trends among the varieties under salinity condition. A total of 55 PS species and 4 LPS species were identified in this study (**Figure 2A**). Salt stress generally triggered an apparent increase in PS species containing long-chain polyunsaturated fatty acids [very long-chain polyunsaturated fatty acids (VLCPUFAs)] in Xu 22 leaves, including PS (20:5/22:6), PS (22:6/15:1), PS (22:1/22:4), LPS (20:5), PS (19:0/20:5), and PS (22:6/19:0). This trend was not observed in Xu 32. The predominant PS species in salinized Xu 22 leaves was PS (20:5/22:6), and its relative abundance increased from 5% in total PS detected under control condition to 75% in total PS detected under salinity condition (**Figure 2B**).

**Figure 3:**
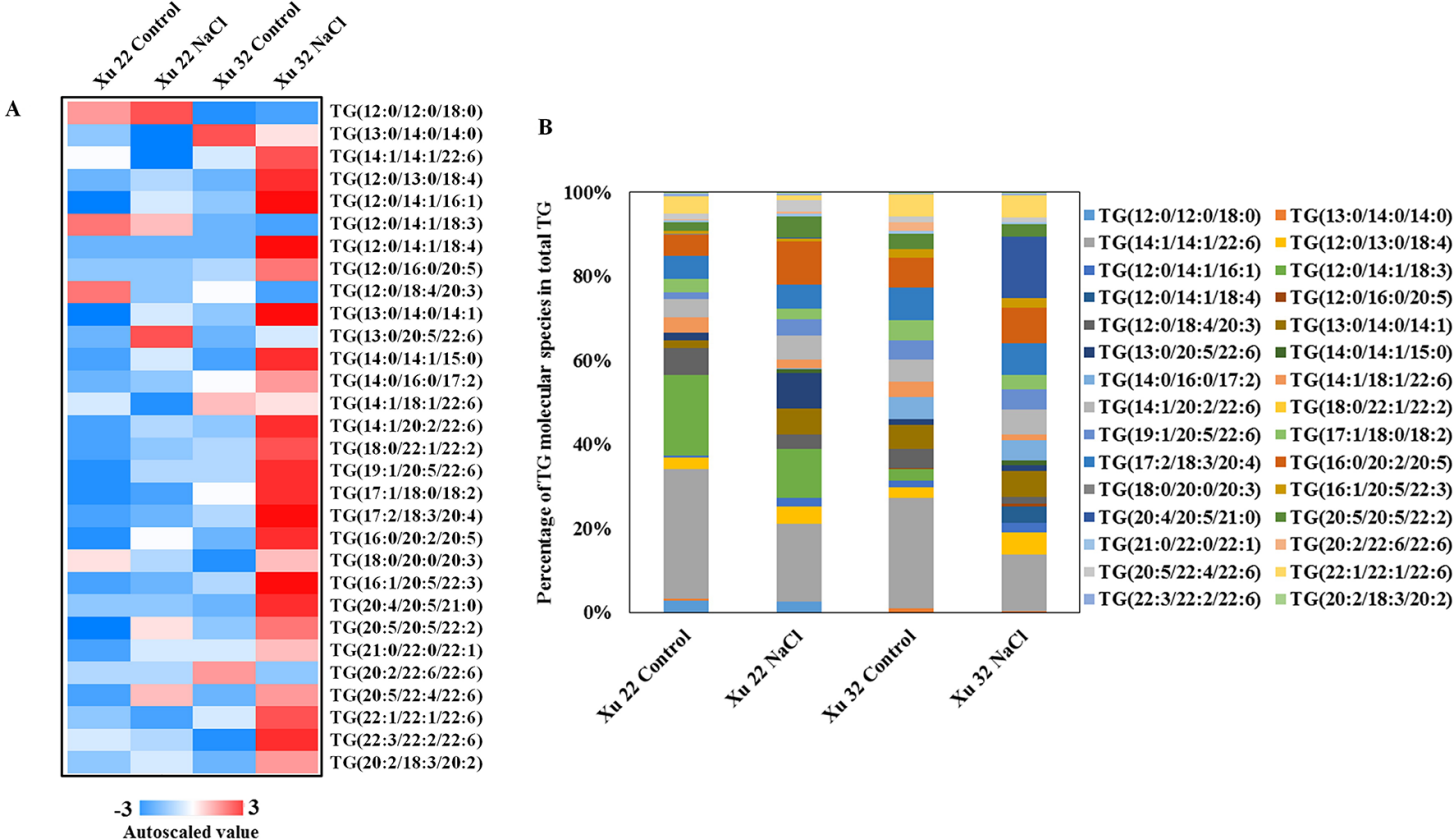
**(A)** Heat map of average autoscaled data of TG molecular species in the leaves of sweet potato cultivars (Xu 22 and Xu 32) under control and NaCl treatment conditions. Autoscaling allows for easy comparison of lipid levels in different samples. The autoscaled value of a lipid in a sample is calculated as follows: [(amount of lipid in a sample) – (average amount of the lipid among all samples)]/(standard deviation of the lipid amount among all samples). **(B)** Relative abundance of TG molecular species. Each TG molecular species is expressed as a percentage of the total amount of TG detected.

Salinity stress significantly increased the abundance of most detected TG species in Xu 32 leaves (23 of the 30 TG species). However, only one TG species increased in NaCl-stressed Xu 22 leaves unlike in the control leaves (**Figure 3A**). The predominant TG species in salinized Xu 32 leaves was TG (20:4/20:5/21:0), and its relative abundance increased from 0% under control condition to 14% in total TG detected under salinity condition (**Figure 3B**). The change trend of TG species in the roots was similar to that in the leaves (**Supplementary Figure S4**).

**Figure 4:**
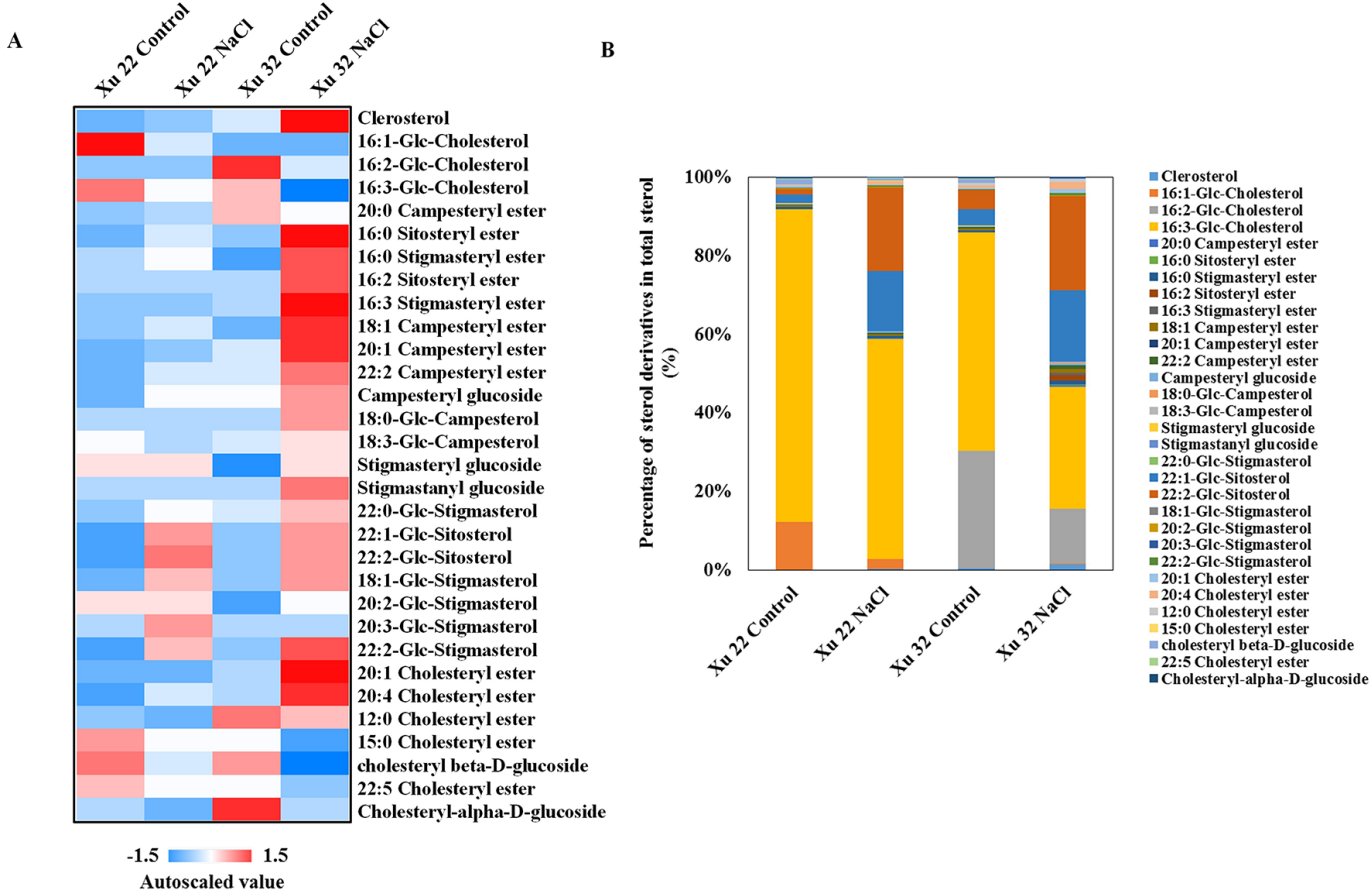
**(A)** Heat map of average autoscaled data of sterol derivatives in the leaves of sweet potato cultivars (Xu 22 and Xu 32) under control and NaCl treatment conditions. Autoscaling allows for easy comparison of lipid levels in different samples. The autoscaled value of a lipid in a sample is calculated as follows: [(amount of lipid in a sample) – (average amount of the lipid among all samples)]/(standard deviation of the lipid amount among all samples). **(B)** Relative abundance of sterol derivatives. Each sterol derivative is expressed as a percentage of the total amount of sterol derivatives detected.

In this study, several sterol derivatives, including free sterol (cholesterol), sterol glycosides, acylated sterol glycosides (ASGs), and sterol esters, were detected in sweet potato (**Figure 4A**). The predominant sterol derivatives in Xu 32 leaves under control condition were 16:2-Glc-Cholesterol and 16:3-Glc-Cholesterol (**Figure 4B**). Salinity stress drastically reduced the amounts of 16:2-Glc-Cholesterol and 16:3-Glc-Cholesterol by 73 and 60%, respectively. The relative abundance of 16:2-Glc-Cholesterol and 16:3-Glc-Cholesterol in total sterols was reduced by salt from 29 to 14% and from 55 to 31%, respectively (**Figure 4B**). In the Xu 22 leaves, the major sterol derivative was 16:3-Glc-Cholesterol (79% of total sterols). Salt stress decreased this ASG from 79 to 55%. In addition, the amounts of two other ASGs (22:1-Glc-Sitosterol and 22:2-Glc-Sitosterol) significantly increased under salinity condition in both varieties (**Figure 4B**). The relative abundance of 22:1-Glc-Sitosterol and 22:2-Glc-Sitosterol increased from 4 to 37% in Xu 22 and from 9 to 42% in Xu 32 after salt stress (**Figure 4B**). These results indicate that the salt-induced change trend of major sterol derivatives was similar in the two sweet potato varieties. Thus, we focused on the roles of PS and TG in mediating salt-defensive responses in sweet potato leaves.

### Exogenous PS Alters Salt Responses in Detached Xu 32 Leaves

**Figure 5:**
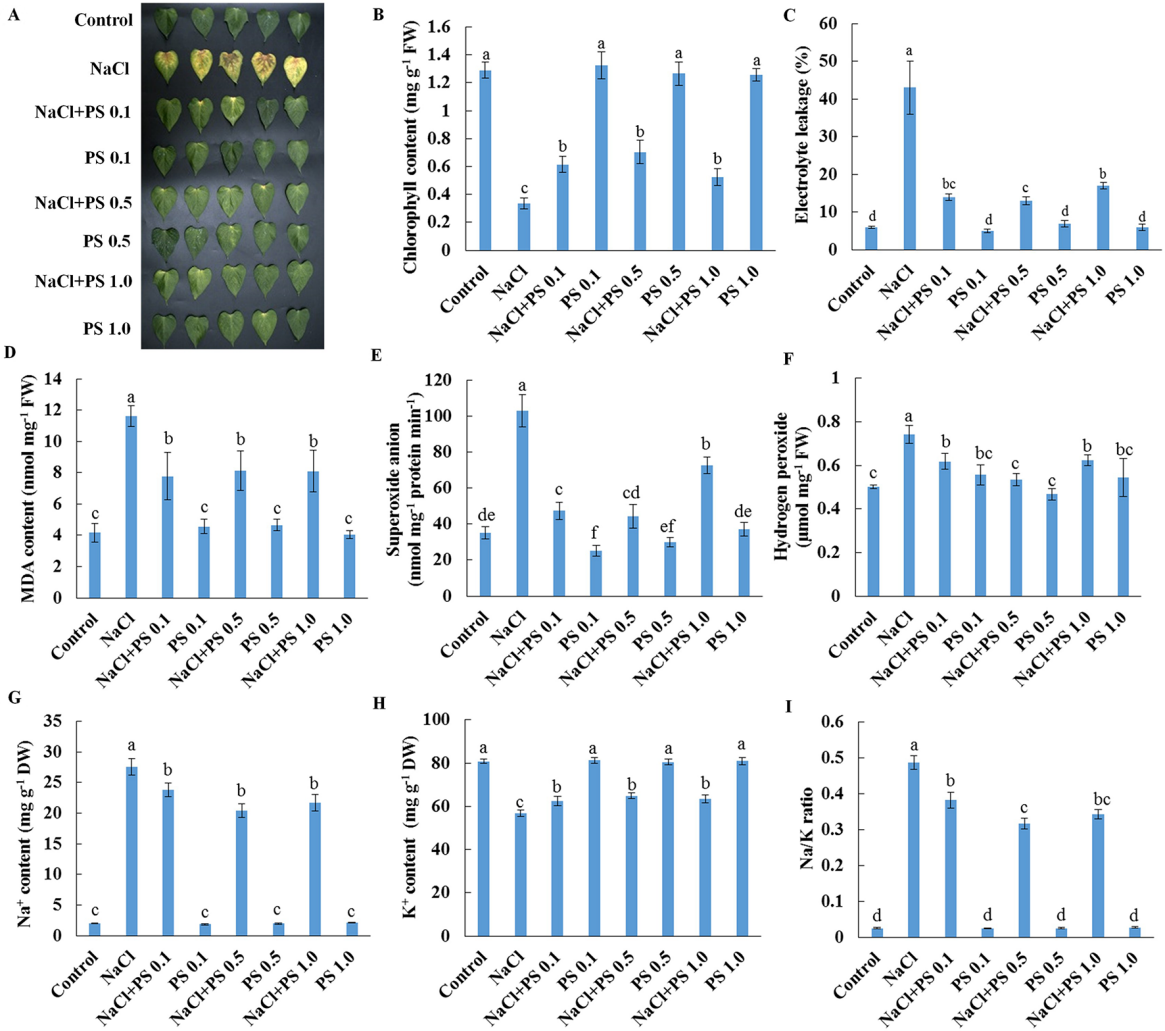
Effects of exogenous PS on physiological responses of detached Xu 32 leaves in the absence or presence of NaCl stress. Detached Xu 32 leaves were pre-treated with various concentrations of PS for 24 h and then subjected to 200-mM NaCl treatment for 6 days. **(A)** Phenotypes of Xu 32 leaves after various treatments. **(B)** Chlorophyll content. **(C)** Electrolyte leakage. **(D)** Malondialdehyde content. **(E)** Superoxide anion production ratio. **(F)** Hydrogen peroxide content. **(G** and **H)** Na^+^ and K^+^ contents. **(I)** Na/K ratio. Columns represent the means of at least five replicates per treatment, and bars represent the standard error of the mean. Columns labeled with different letters (a–f) indicate significant difference at *P* < 0.05.

We next used detached leaves of Xu 32 and exogenous application of PS (extracted from soybean) as experimental model for further analysis of the role of PS in mediating salt responses. Salt treatment (200-mM NaCl) for 6 days triggered an evident senescence of Xu 32 leaves, as indicated by distinct leaf chlorosis (**Figure 5A**); markedly decreased chlorophyll content (**Figure 5B**); sharply increased membrane permeability (**Figure 5C**), membrane lipid peroxidation (**Figure 5D**), and reactive oxygen species (ROS) accumulation (**Figures 5E, F**); drastically increased Na^+^ accumulation (**Figure 5G**); decreased K^+^ abundance (**Figure 5H**); substantially elevated cellular Na^+^/K^+^ ratio (**Figure 5I**). We tested three PS concentrations (0.1, 0.5, and 1.0 µM) and observed that all PS doses alleviated salt-induced leaf chlorosis (**Figure 5A**) and partially recovered the salt-reduced chlorophyll content (**Figure 5B**). The salt-triggered accumulation of ROS was significantly inhibited by exogenous PS and thus caused the reduction of membrane lipid peroxidation and membrane permeability (**Figures 5C–F**). Furthermore, PS significantly reduced the Na^+^/K^+^ ratio in salt-stressed Xu 32 leaves mainly through the inhibitory effect of PS on salt-induced Na^+^ buildup and K^+^ loss (**Figures 5G–I**). This alleviatory effect of PS on salt-induced leaf senescence was also observed in PS extracted from porcine brain, which contained 22:6 fatty acid chains (data not shown).

**Figure 6:**
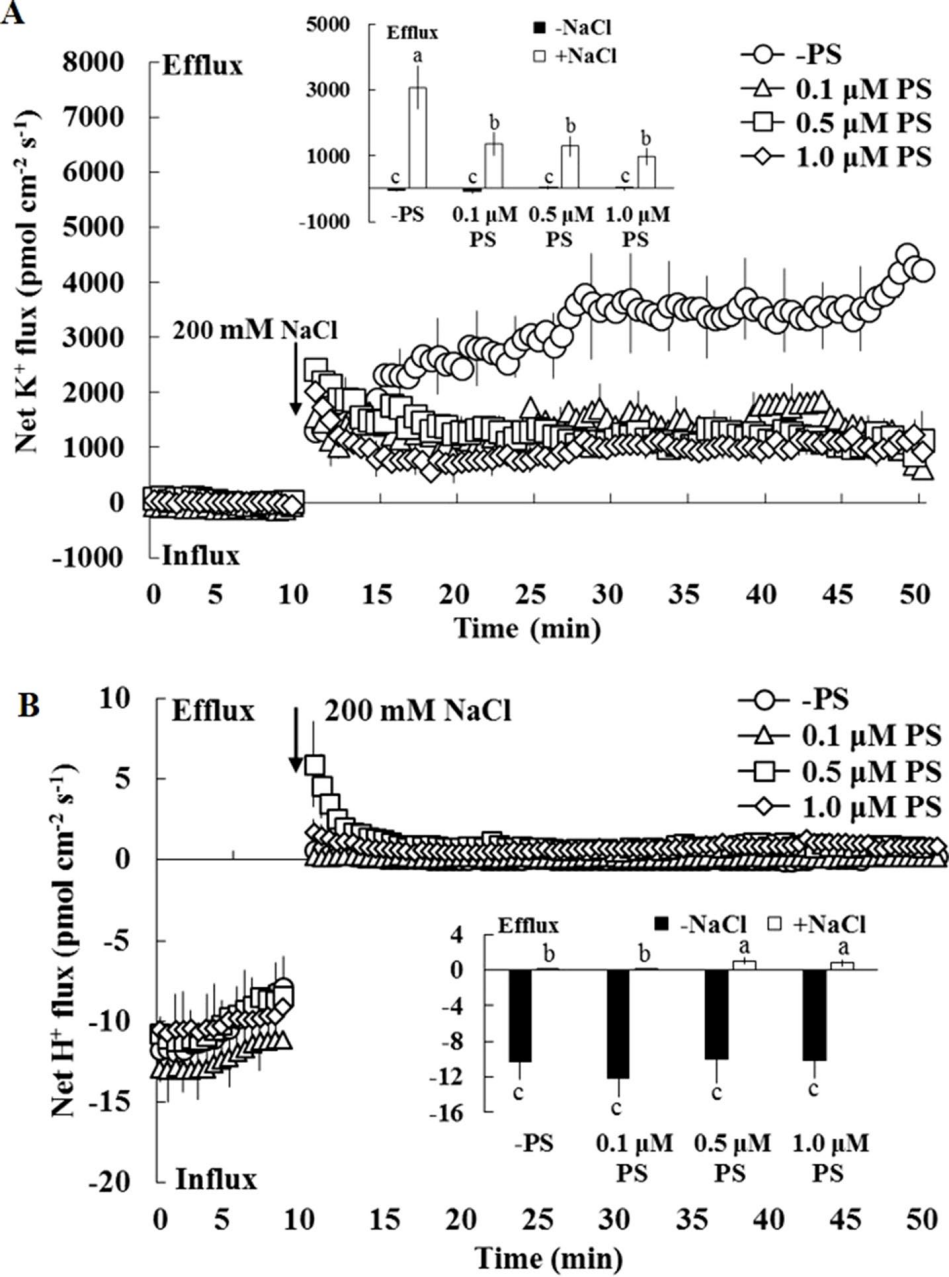
Effects of exogenous PS on salt-induced transient K^+^ **(A)** and H^+^ **(B)** fluxes in mesophyll cells of Xu 32. Detached Xu 32 leaves were pre-treated with various concentrations of PS for 24 h, and transient ion fluxes in mesophyll cells were recorded. Before the NaCl shock, steady fluxes of K^+^ and H^+^ were monitored for 10 min as control. Each point is the mean of 10 individual leaves, and bars represent the standard error of the mean. Inserted panels show the mean fluxes of K^+^ and H^+^ before and after NaCl shock. Different letters (a–c) denote significant differences at *P* < 0.05.

The earlier results indicate that PS contributes to the regulation of ion transport in salinized Xu 32 leaves. Thus, we next examined the effect of exogenous PS on salt-triggered K^+^ and H^+^ fluxes in Xu 32 mesophyll tissue. NMT data showed that salt shock (200-mM NaCl) significantly enhanced K^+^ efflux, which reached 3,000 pmol cm^–2^ s^–1^ after salt exposure, in Xu 32 mesophyll tissue (**Figure 6A**). All the tested doses of PS alleviated salt shock-triggered K^+^ efflux by at least 60% (**Figure 6A**). The mesophyll tissue exhibited a net H^+^ influx before the salt shock. NaCl addition caused a slight shift in H^+^ influx toward an efflux, and exogenous PS (0.5 and 1.0 µM) reinforced this salt-induced H^+^ efflux in Xu 32 mesophyll tissue (**Figure 6B**). PS exerted no effect on net K^+^ and H^+^ fluxes without the addition of NaCl shock (**Figure 6**).

**Figure 7:**
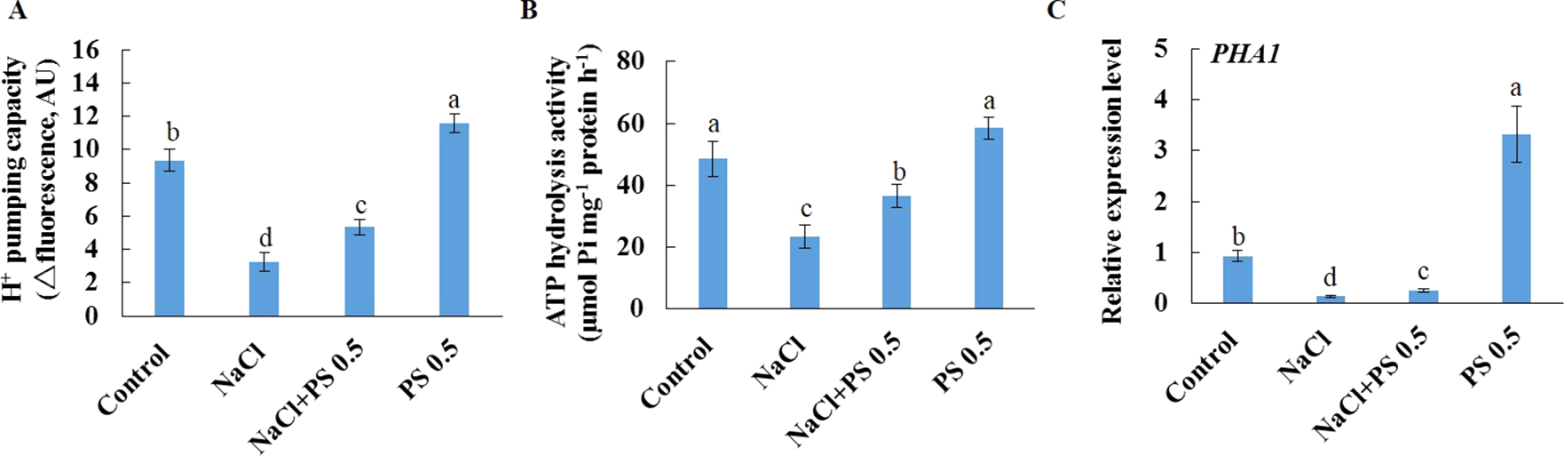
Effects of exogenous PS on PM H^+^-ATPase activity and *PHA1* expression level in detached Xu 32 leaves in the absence or presence of NaCl treatment. Detached Xu 32 leaves were pre-treated with 0.5-µM PS for 24 h and subjected to 200-mM NaCl treatment for 6 days. **(A)** H^+^ pumping activity as indicated by the ACMA fluorescent quenching method in PM vesicles purified from various groups of leaf samples. **(B)** ATP hydrolysis activity as indicated by the Pi-releasing method in PM vesicles purified from various groups of leaf samples. **(C)** *PHA1* expression level in various groups of leaf samples. Each column is the mean of three replicates, and bars represent the standard error of the mean. Columns labeled with different letters (a–d) indicate significant difference at *P* < 0.05.

We further purified PM vesicles from different treatment groups of Xu 32 leaves, followed by an H^+^-ATPase activity assay using an ACMA-based method and a Pi releasing-based method. PM vesicles purified from PS (0.5 µM)-treated leaves exhibited higher PM H^+^-pumping capacity than the control group, as indicated by the difference in ACMA fluorescence quenching after H^+^-ATPase activation by Mg^2+^ (**Figure 7A**). Salt treatment markedly reduced the PM H^+^-pumping capacity of Xu 32 leaves. However, exogenous PS treatment partially reduced the salt-inhibited PM H^+^-pumping capacity of Xu 32 leaves (**Figure 7A**). The ATP hydrolysis activity of PM vesicles isolated from salt-stressed Xu 32 leaves sharply decreased relative to the control group. However, PS alleviated the inhibitory effect of salt on the PM ATP hydrolysis activity. PS treatment exerted no influence on the PM ATP hydrolysis activity in the absence of NaCl stress (**Figure 7B**). Moreover, exogenous PS treatment significantly enhanced the expression level of *PHA1*, which encodes PM H^+^-ATPase in sweet potato, in the absence or presence of NaCl treatment (**Figure 7C**).

### Inhibition of TG Mobilization Alters Salt Responses in Detached Xu 22 Leaves

**Figure 8:**
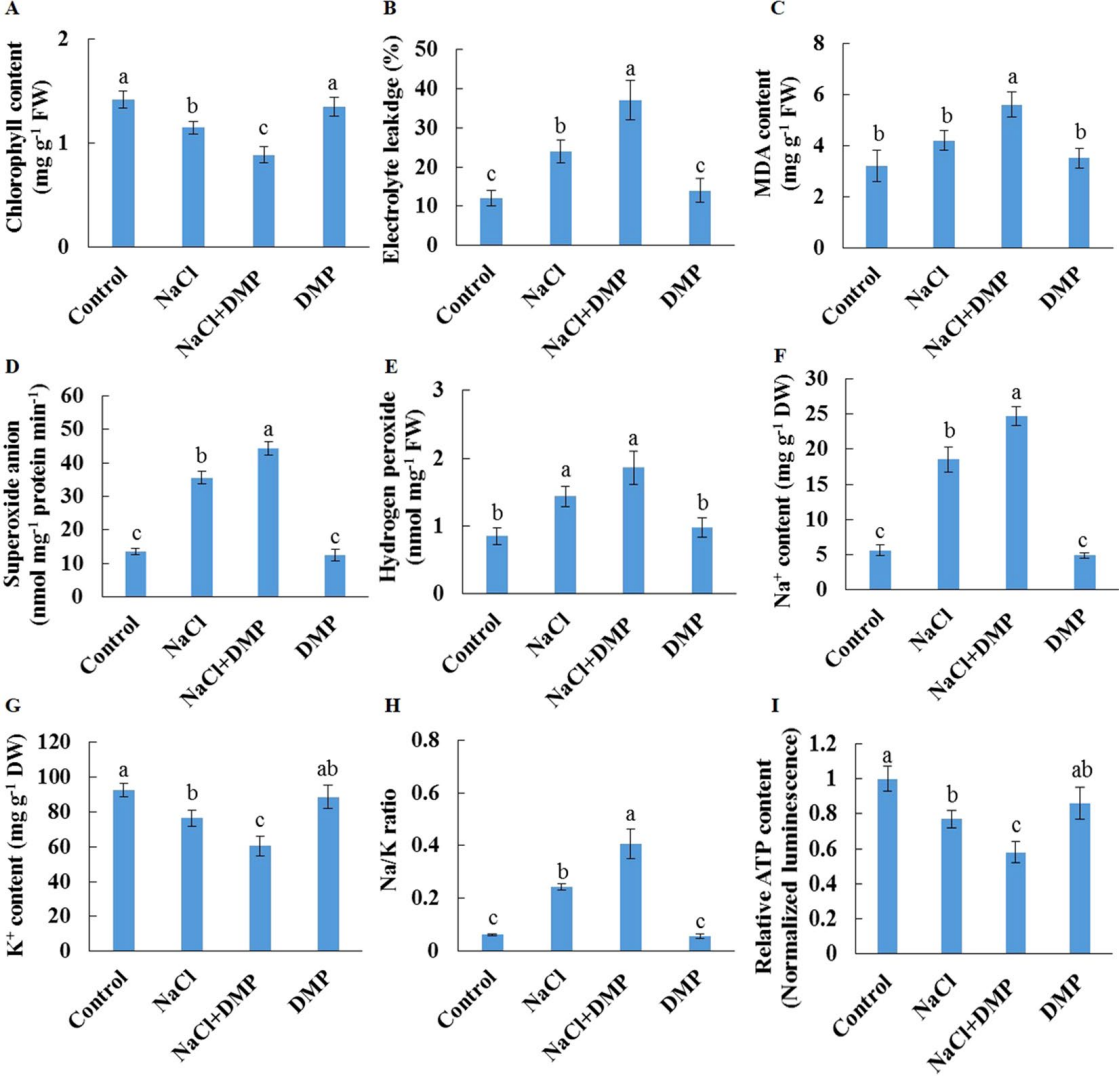
Effects of DMP on physiological responses of detached Xu 22 leaves in the absence or presence of NaCl stress. Detached Xu 22 leaves were pre-treated with 25-µM DMP for 24 h and then subjected to 200-mM NaCl treatment for 6 days. **(A)** Chlorophyll content. **(B)** Electrolyte leakage. **(C)** Malondialdehyde content. **(D)** Superoxide anion production ratio. **(E)** Hydrogen peroxide content. **(F** and **G)** Na^+^ and K^+^ contents. **(H)** Na/K ratio. **(I)** Relative ATP level. Columns represent the means of at least five replicates per treatment, and bars represent the standard error of the mean. Columns labeled with different letters (a–f) indicate significant difference at *P* < 0.05.

**Figure 9:**
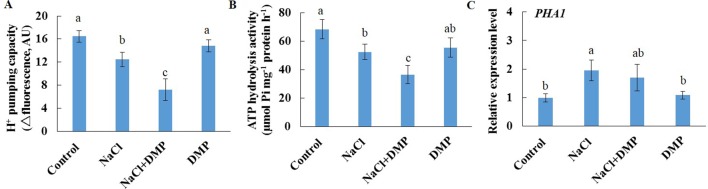
Effects of DMP on PM H^+^-ATPase activity and *PHA1* expression level in detached Xu 22 leaves in the absence or presence of NaCl treatment. Detached Xu 22 leaves were pre-treated with 25-µM DMP for 24 h and then subjected to 200-mM NaCl treatment for 6 days. **(A)** H^+^ pumping activity as indicated by the ACMA fluorescent quenching method in PM vesicles purified from various groups of leaf samples. **(B)** ATP hydrolysis activity as indicated by the Pi-releasing method in PM vesicles purified from various groups of leaf samples. **(C)** *PHA1* expression level in various groups of leaf samples. Each column is the mean of three replicates, and bars represent the standard error of the mean. Columns labeled with different letters (a–c) indicate significant difference at *P* < 0.05.

Salt stress decreased the expression level of *SDP1*, which encodes a lipase required for the breakdown of TG (Eastmond, 2006), in Xu 32 leaves (**Supplementary Figure S5**). However, salinity stress in Xu 22 leaves showed no influence on the expression level of *SDP1* (**Supplementary Figure S5**). The expression level of two acyltransferases (*PDAT1* and *DGAT1*) that are required for TG synthesis showed no significant difference between the two cultivars in most tested time points in the presence of NaCl stress (**Supplementary Figure S5**). These results indicate that compared with Xu 32 leaves, Xu 22 leaves may feature higher capacity of TG breakdown under salinity condition and thus contribute to less accumulation of TG (**Figures 1** and **3**). To investigate the role of TG in the responses of sweet potato leaves to salt stress, we used DMP, which is an inhibitor of TG mobilization in plants (Brown et al., 2013; McLachlan et al., 2016; Yu et al., 2018), in further experiments. In this series of experiments, detached Xu 22 leaves were selected as experimental model. DMP (25 µM) treatment enhanced lipid droplet accumulation in Xu 22 leaves under control and salinity conditions as indicated by the BODIPY 493/503-specific fluorescence (our unpublished data). This result suggests that DMP can inhibit TG breakdown in sweet potato leaves. The physiological responses of Xu 22 leaves to salinity stress were impaired in the presence of DMP, as revealed by the decreased chlorophyll content, K^+^ abundance, and cellular ATP level (**Figures 8A, G, I**); and increased membrane permeability (**Figure 8B**), membrane lipid peroxidation (**Figure 8C**), ROS accumulation (**Figures 8D, E**), Na^+^ content, and Na^+^/K^+^ ratio (**Figures 8F–H**). DMP treatment showed no influence on these physiological indices in the absence of NaCl stress (**Figure 8**). Furthermore, DMP treatment significantly reduced PM H^+^-ATPase activity in salinized Xu 22 leaves but not in the control group (**Figures 9A, B**). However, DMP caused no effect on the expression of *PHA1* under control or salinity conditions (**Figure 9C**).

## Discussion

In this study, untargeted lipidome analyses were performed to identify lipid classes or specific lipid species that were regulated during salt stress in two sweet potato cultivars. Salt stress affects plant roots directly. Thus, most previous studies focused on salt-induced lipid remodeling of the roots of different plant species (Mansour et al., 2015). Here, a moderate alteration of root lipidome was triggered by salt stress in two sweet potato varieties (**Supplementary Figure S2**). Under salinity condition, salt-tolerant Xu 22 maintained higher PC and PE abundance in the roots than salt-sensitive Xu 32 (**Supplementary Figure S2**). This trait is consistent with previous reports and may help Xu 22 sustain PM integrity and maintain the proper functioning of several membrane proteins in salinized roots (Mansour et al., 2015; Natera et al., 2016). We observed a more evident remodeling of lipidome in leaves (**Figure 1**). Several interesting lipid classes or specific lipid species were discovered in the responses of leaves to salinity stress, and the potential roles of these lipids in mediating tissue tolerance upon salinity stress in Xu 22 were further discussed.

PS is an abundant negatively charged phospholipid of biological membranes in both prokaryotic and eukaryotic cells; it is involved in various biological processes, for example, as an enzyme cofactor and recruiter of signaling molecules (Vance and Steenbergen, 2005). Phosphatidylserine synthase (PSS), which encodes a base-exchange-type PS synthase, has been recently identified in *Arabidopsis* and rice plants (Yamaoka et al., 2011; Yin et al., 2013). PSS-dependent PS synthesis is essential for developmental processes, including the maturation of microspore (Yamaoka et al., 2011), development of inflorescence meristem and organs (Liu et al., 2013), and cell elongation in the uppermost internode of rice (Ma et al., 2016). In the present study, PS species containing VLCPUFAs were significantly enhanced by salinity stress in Xu 22 leaves (**Figures 1** and **2**). This result suggests that PS contributes to tissue tolerance upon salinity stress in Xu 22 leaves. Another evidence is the distinctly delayed salt-induced leaf senescence in Xu 32 caused by exogenous PS (**Figure 5**). However, we are unsure whether the beneficial role of exogenous PS originates from the outside or inside of the cells. Several potential roles of PS in alleviating NaCl toxicity were postulated.

K^+^ retention in leaf mesophyll cells is an important salt-tolerant trait in different plants, including glycophytes (barley and wheat) and halophytes (Wu et al., 2015; Percey et al., 2016; Zhu et al., 2016). The different capacities of K^+^ retention under salinity stress were determined by controlling K^+^ loss through depolarization-activated K^+^ outward-rectifying channel (DA-KORC) and ROS-activated non-selective cation channel (NSCC) (Wu et al., 2015). Maintaining high PM H^+^-ATPase activity and low ROS level under salinity condition contributes to less K^+^ loss *via* DA-KORC and ROS-activated NSCC (Chen et al., 2007; Sun et al., 2009b; Bose et al., 2015; Chakraborty et al., 2016). Previous studies discovered that using *in vitro* experimental system PS promotes PM H^+^-ATPase activity in various plant species (Morales-Cedillo et al., 2015). In the present study, exogenous PS enhanced PM H^+^-ATPase activity and reduced ROS accumulation in salinized Xu 32 leaves (**Figures 5** and **7**). This phenomenon alleviated K^+^ efflux *via* DA-KORC or ROS-activated NSCC, which improved cellular K^+^/Na^+^ homeostasis in salinized Xu 32 leaves (**Figures 5** and **6**). Although no evidence of PS localization was observed within the cells, the high amount of PS in PM, as estimated by the total enhanced amount of PS, possibly improved the PM H^+^-ATPase activity in salinized Xu 22 leaf cells. Thus, salt-enhanced PS abundance or exogenous application of PS maintains K^+^/Na^+^ homeostasis *via* activating the PM H^+^-ATPase activity in sweet potato leaves.

Salinity stress generally impairs the integrity of PM. PM resealing is a necessary response that allows cells to survive membrane disruption caused by high salinity (Schapire et al., 2008). In *Arabidopsis*, repairing of PM after salt injury is mediated by synaptotagmin 1, which is a Ca^2+^-activated membrane fusion protein that facilitates the delivery of intracellular membranes to wound sites by a mechanism resembling that in animal cells (Schapire et al., 2008). In animals, PS binds to synaptotagmin I in a Ca^2+^-dependent manner, and this interaction plays an important role in exocytosis (Zhang et al., 2009). In rice, PSS-dependent PS synthesis is also required for exocytosis (Ma et al., 2016). Therefore, we can infer that the increment in PS abundance in salinized sweet potato leaves promotes exocytosis and PM resealing and thus contributes to the PM integrity of leaves under salinity stress.

PS is a negatively charged phospholipid; maintaining high PS abundance in PM may contribute to electrostatic interaction with other important proteins (Stace and Ktistakis, 2006). For example, in animals, PS binds to phospholipase C, which hydrolyses PIP_2_ to form IP_3_, which is an important signaling molecule in stimulating internal Ca^2+^ release in animals and plants (Singh et al., 2015), through serine-headgroup specific electrostatic interactions (Stace and Ktistakis, 2006). In addition, homologous animal PS-binding proteins, including annexin, protein kinase C, diacylglycerol kinase, NO synthase, and synaptotagmin, are reportedly involved in the mediation of plant salt tolerance (Zhao et al., 2007; Schapire et al., 2008; Laohavisit et al., 2013; Golldack et al., 2014; Meringer et al., 2016). Recently, variations in PS levels in the PM have been reported as a physiological regulator of small GTPase signaling during plant development (Platre et al, 2019). Thus, we suggest that the enhancement of PS abundance may contribute to the recruitment of important proteins required for salt tolerance in salinized sweet potato leaves. In addition, exogenous application of PS may trigger unknown signaling events that enhance tissue tolerance to salinity stress. However, these assumptions still need further experiments for confirmation.

TG is the major reserve of FAs for carbohydrates and energy production during seed germination and early seedling establishment; it is also essential for normal growth and development of adult plants (Xu and Shanklin, 2016). Although vegetative tissues accumulate minor levels of TG, this storage lipid serves multiple important roles in plant responses to environmental factors (Abida et al., 2015; Mueller et al., 2015). TG functions as a buffer for cytotoxic FAs and other lipid intermediates, thereby playing a key role in intracellular lipid homeostasis and cell survival (Fan et al., 2013; Fan et al., 2014). Under high-temperature condition, elevated TG serves as a transient storage for FAs that may be required for membrane remodeling during heat acclimation (Mueller et al., 2015). However, the massive accumulation of TG is frequently observed in senescing leaves of plants (Watanabe et al., 2013; Kim et al., 2015). In the present study, the abundance of salt-increased TG was significantly higher in Xu 32 leaves than that in salt-tolerant Xu 22 (**Figures 1** and **3**). Thus, we suggest that salt-triggered TG accumulation in Xu 32 leaves is a hallmark of senescence and reflects the low salt resistance of plants. The minor accumulation of TG in salinized Xu 22 leaves may be ascribed to high lipase gene (*SDP1*) expression and the corresponding lipase activity (**Supplementary Figure S5**). Following TG hydrolysis by lipases, FAs are transported and enter peroxisomes for β-oxidation and energy turnover (Xu and Shanklin, 2016). A recent study has reported that the ATP derived from TG breakdown and β-oxidation participates in the activation of PM H^+^-ATPase during blue light-triggered stomatal opening (McLachlan et al., 2016). In the present study, the inhibition of TG breakdown weakened the PM H^+^-ATPase activity, cellular energy state, and tissue tolerance in salinized Xu 22 leaves (**Figures 8** and **9**). These results show that high capacity of TG breakdown and energy turnover in the leaves contribute to tissue tolerance upon salinity stress in sweet potato.

Sterol lipids play crucial roles during adaption to abiotic stresses and plant–pathogen interactions (Wewer et al., 2011). Maintenance of free sterol abundance in the PM is important for regulating membrane fluidity, permeability, and transporter protein activity in salinized plants (Mansour et al., 2015). Cholesterol stimulates PM H^+^-ATPase activity in soybean (Grandmougin-Ferjani et al., 1997; Rossard et al., 2010). However, in the present study, salt stress enhanced cholesterol abundance in salt-sensitive Xu 32 leaves but not in salt-tolerant Xu 22 (**Figure 4**). In addition, PM H^+^-ATPase activity was higher in Xu 22 leaves than that in Xu 32 under salinity condition (**Figures 7** and **9**). Considering the stimulating role of PS on the PM H^+^-ATPase activity (**Figure 7**), we suggest that the salt-enhanced PS is required for the maintenance of PM H^+^-ATPase activity in Xu 22 leaves. The salt-enhanced cholesterol abundance is correlated to the maintenance of basal PM H^+^-ATPase activity in salinized Xu 32 leaves. Thus, cholesterol provided minimal contributions to the higher PM H^+^-ATPase activity and improved tissue tolerance of Xu 22 leaves under salinity stress.

The abundance of campesterol and stigmasterol in the PM is enhanced in halophyte callus and salt-tolerant crops (Kerkeb et al., 2001; Wu et al., 2005). Stigmasterol stimulates PM H^+^-ATP activity in sugar beet leaves (Rossard et al., 2010). These results imply that these free sterols play crucial roles in stabilizing membrane structure and mediating ion transport. In the present study, salt stress increased the abundance of sterol esters (mainly campesteryl and stigmasteryl esters) in Xu 32 leaves (**Figure 4A**), indicating the salt-enhanced esterification of these free sterols and consequent decrease in the abundance of the corresponding free sterols. These phenomena may disturb the membrane structure stability and PM H^+^-ATPase activity in salinized Xu 32 leaves. Thus, salt-enhanced sterol esters could be potential biomarkers of salt sensitivity in sweet potato. This viewpoint must be verified by further studies on several varieties with varying degrees of salt tolerance.

SGs and ASGs are ubiquitous compositions of cells in vascular plants and function as membrane components, storage forms of sterols, and signaling molecules (Grille et al., 2010). Increased SG synthesis *via* the overexpression of the gene that encodes sterol glycosyltransferase from *Withania somnifera* enhances salt tolerance in transgenic tobacco plants (Pandey et al., 2014). Compared with the salt-susceptible cultivar Xu 32, the salt-tolerant Xu 22 may be more efficient in mediating ASG level in the leaves to maintain membrane bilayer structure under salinity stress. In the present study, Xu 22 leaves maintained higher relative abundance of 16:3-Glc-Cholesterol, 22:1-Glc-Sitosterol, and 22:2-Glc-Sitosterol (92% in total sterol derivatives) than Xu 32 leaves (77% in total sterol derivatives) under salinity condition (**Figure 4**). Higher ASG abundance may provide more sugar head groups to membranes and thus contribute to resistance against the osmotic effects caused by salt stress (Tarazona et al., 2015). This result suggests that ASGs are potential biomarkers of salt tolerance in sweet potato. However, the detailed function of ASGs in plant salt tolerance still needs further investigation.

In summary, this study demonstrates the detailed changes in the roots and leaves lipidome of two sweet potato cultivars under salinity stress. PS synthesis and TG mobilization play important roles in mediating salt-defensive responses in sweet potato leaves. In addition, exogenous application of PS on leaves can alleviate the tissue toxicity of NaCl, and this priming method may be useful in the field. However, future studies should elucidate the roles of specific lipid species in the mediation of plant salt tolerance, particularly the genes that contribute to the synthesis of PS species containing VLCPUFAs during salinity stress and the proteins that interact with PS and contribute to salt tolerance in plants.

## Data Availability

All datasets generated for this study are included in the manuscript and the **Supplementary Files.**

## Author Contributions

JS and ZL conceived and designed the research; YY, MK, ZG, YL, and YX performed the research and analyzed the data; JS wrote the paper; YJL, QC, and ZT provided the plant materials. All authors carefully read and approved the final manuscript.

## Funding

This work was supported by the National Key R & D Program of China (2018YFD1000704, 2018YFD1000705, 2018YFD1000700), the National Natural Science Foundation of China (31871684, 31771367), Major Projects of Natural Science Foundation of the Higher Education Institutions of Jiangsu Province (18KJA180004), the earmarked fund for China Agriculture Research System (CARS-10-B03), the Priority Academic Program Development of Jiangsu Higher Education Institutions (PAPD) and the Natural Science Foundation of Jiangsu Province (BK20161162).

## Conflict of Interest Statement

The authors declare that the research was conducted in the absence of any commercial or financial relationships that could be construed as a potential conflict of interest.
