# Supplementary material for: Involvement of Phosphatidylserine and Triacylglycerol in the Response of Sweet Potato Leaves to Salt Stress
Source: Front Plant Sci. 2019 Sep 10;10:1086. doi: 10.3389/fpls.2019.01086 (PMC6746921; doi:10.3389/fpls.2019.01086)
Supplement: Supplementary file 5 [file DataSheet_1.pdf]

Supplementary Figures

Supplementary Figure 1

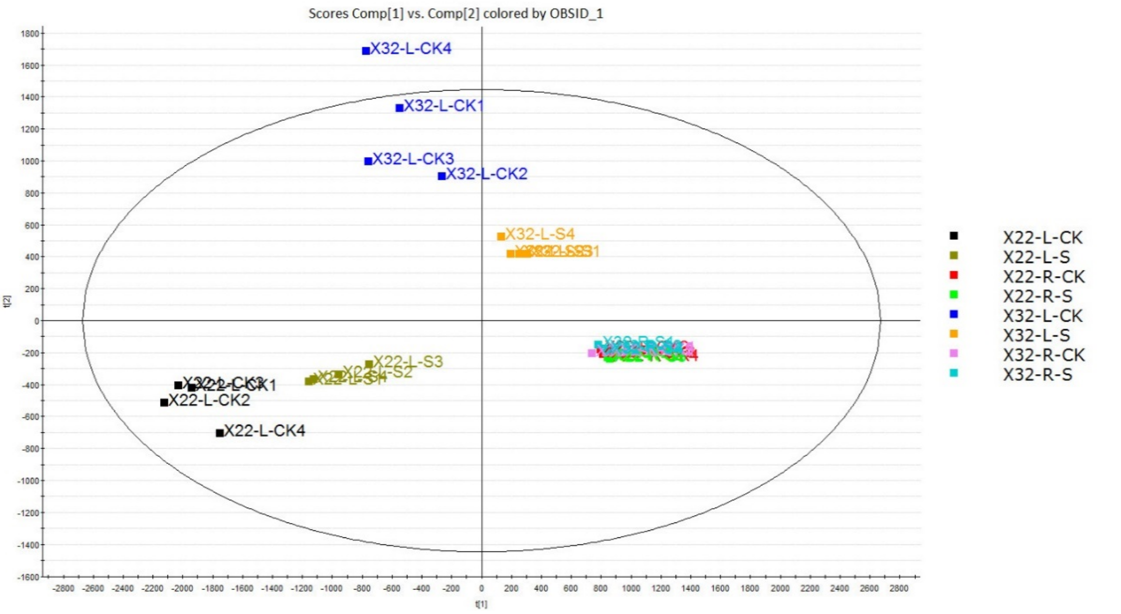

Figure S1 Principal component analysis (PCA) of the identified lipids derived from roots and leaves of control and NaCl-treated (200 mM for 7 days) sweet potato cultivars (Xu 22 and Xu 32). X22-L-CK: leaf samples collected from the control Xu 22; X22-L-S:leaf samples collected from the NaCl-stressed Xu 22; X22-R-CK, root samples collected from the control Xu 22; X22-R-S, root samples collected from the NaCl-stressed Xu 22; X32-L-CK, leaf samples collected from the control Xu 32; X32-L-S, leaf samples collected the NaCl-stressed Xu 32; X32-R-CK, root samples collected from the control Xu 32; X32-R-S, root samples collected from the NaCl-stressed Xu 32.

Supplementary Figure 2

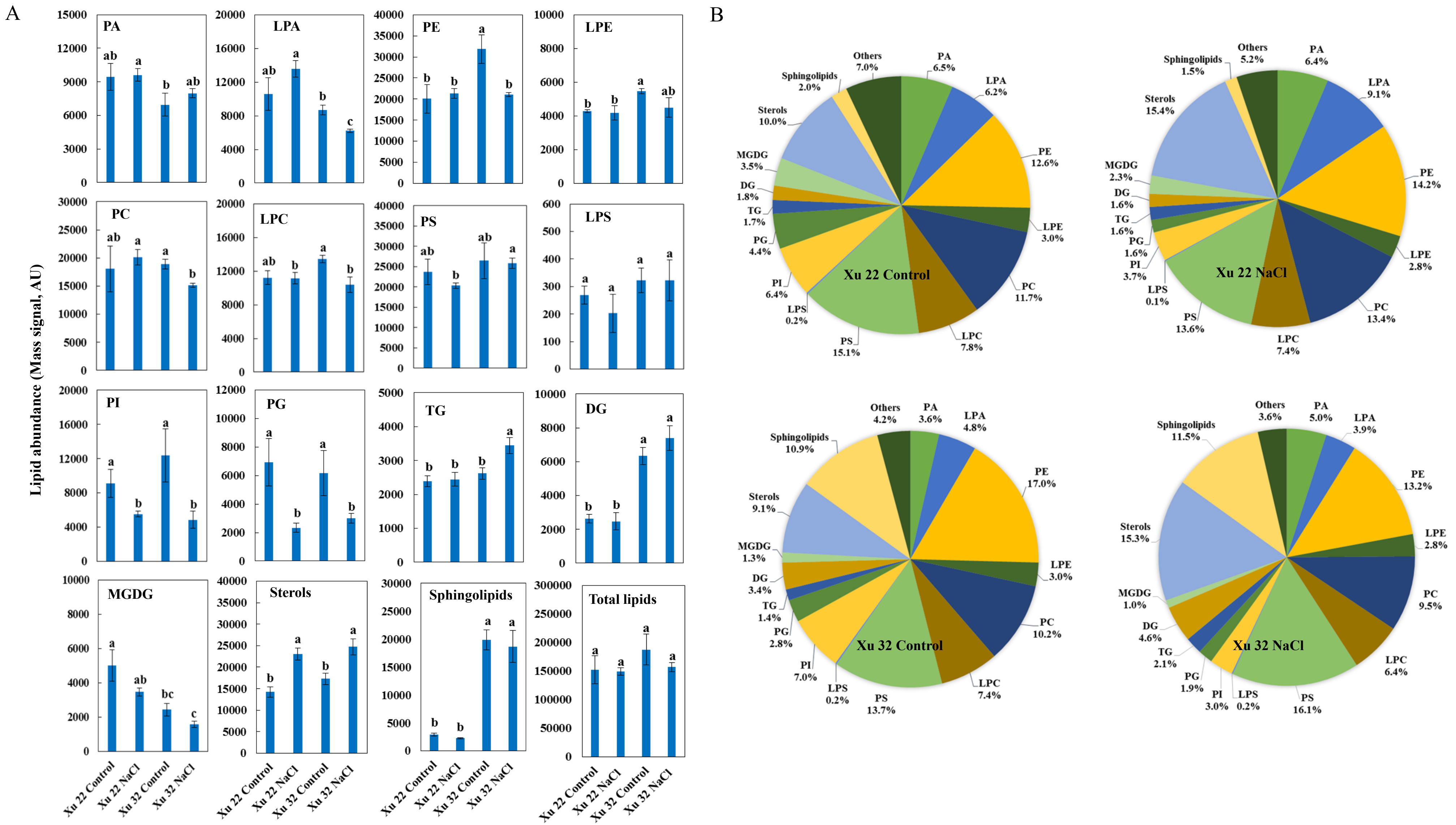

Figure S2 Effects of NaCl stress (200 mM for 7 days) on total amount of lipids in various head group classes of roots in sweet potato cultivars Xu 22 and Xu 32. (A) Columns represent means of four replicates per treatment and bars represent the standard error of the mean. Columns labelled with different letters (a–d) indicate significant difference at $P < 0.05$. (ANVOA). (B) The relative abundance of lipids assigned with the major lipid classes in control and stressed samples at 7 days post of treatment of NaCl. Each lipid class is expressed as a percentage of the total amount of lipids detected. PA, phosphatidic acid; LPA, lysophosphatidic acid; PE, phosphatidylethanolamine; LPE, lysophosphatidylethanolamine; PC, phosphatidylcholine; LPC, lysophosphatidylcholine; PS, phosphatidylserine; LPS, lysophosphatidylserine; PI, phosphatidylinositol; PG, phosphatidylglycerol; TG, triacylglycerols; DG, diacylglycerols; MGDG, monogalactosyldiacylglycerol.

Supplementary Figure 3

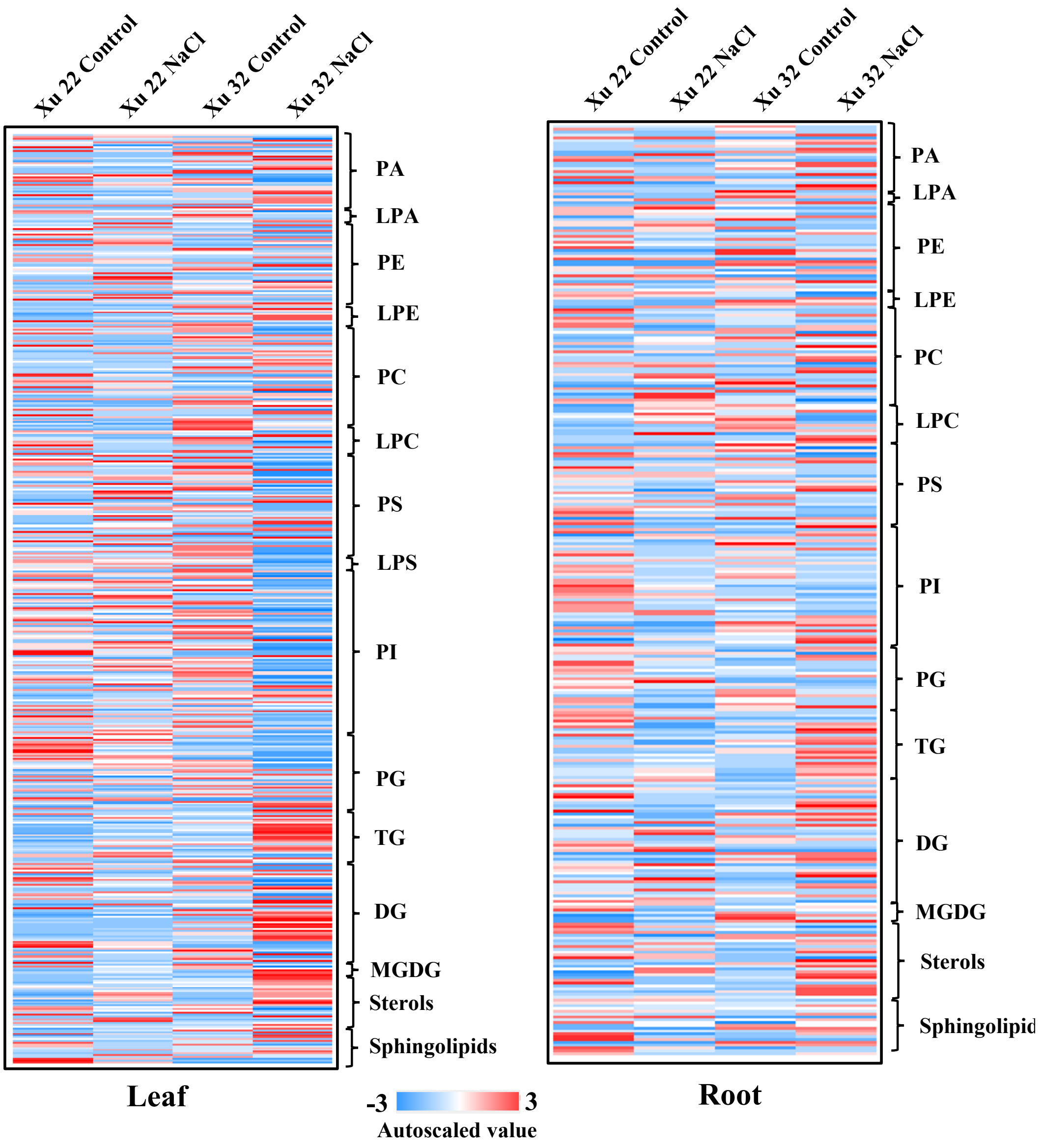

Figure S3 Heat map of average autoscaled data of 524 (leaf) and 330 (root) detected lipid molecular species that belong to 15 major lipid classes in sweet potato cultivars (Xu 22 and Xu 32) under control and NaCl treatment conditions. Autoscaling allows for easy comparison of lipid levels in different samples. The autoscaled value of a lipid in a sample is calculated as follows: [(the amount of lipid in that sample)-(the average amount of that lipid among all samples)] divided by (the standard deviation for the amount of that lipid among all samples).

Supplementary Figure 4

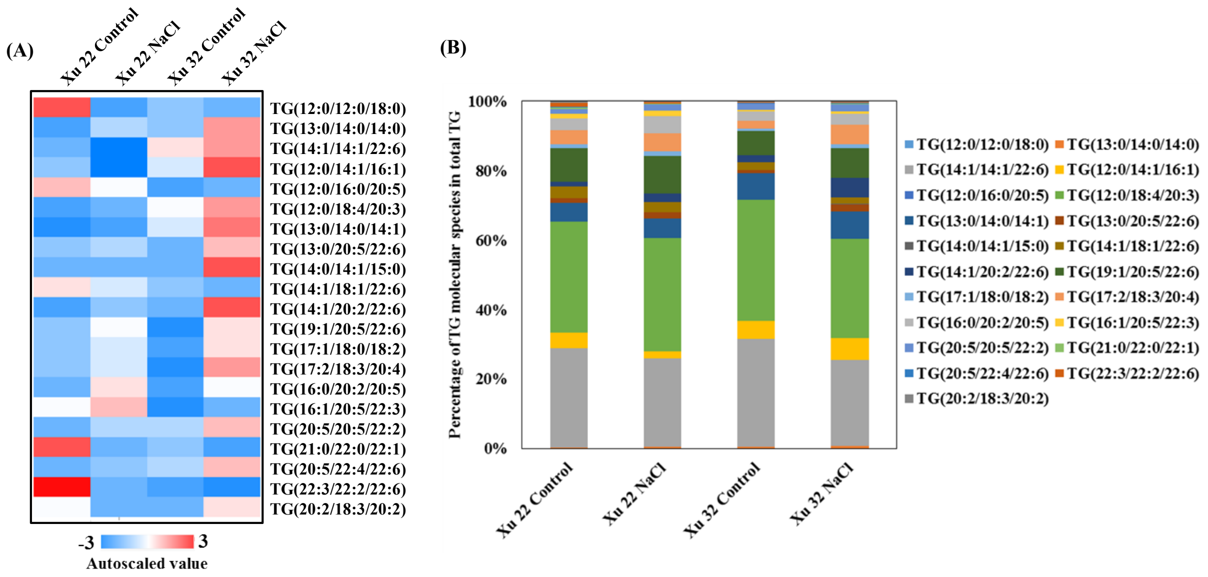

Figure S4 (A) Heat map of average autoscaled data of TG molecular species in the roots of sweet potato cultivars (Xu 22 and Xu 32) under control and NaCl treatment conditions. Autoscaling allows for easy comparison of lipid levels in different samples. The autoscaled value of a lipid in a sample is calculated as follows: [(the amount of lipid in that sample)-(the average amount of that lipid among all samples)] divided by (the standard deviation for the amount of that lipid among all samples). (B) The relative abundance of TG molecular species. Each TG molecular species is expressed as a percentage of the total amount of TG detected.

Supplementary Figure 5

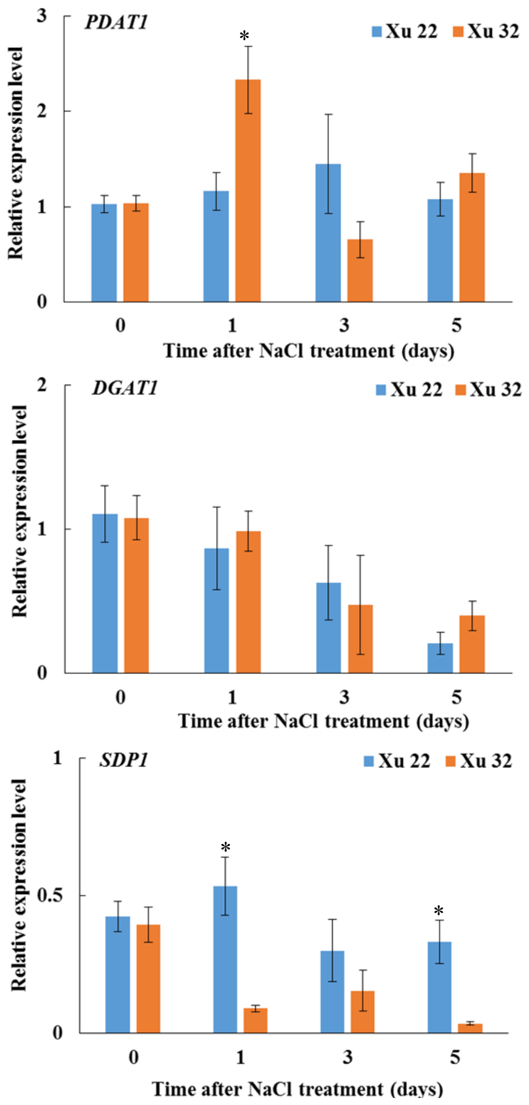

Figure S5 Effects of NaCl stress on the expression level of *PDAT1*, *DGAT1* and *SDP1* in Xu 22 and Xu 32 leaves. Xu 22 and Xu 32 were subjected to the 200 mM NaCl treatment for 1, 3 and 5 days. *PDAT1*, *DGAT1* and *SDP1* expression level were quantified by qRT-PCR. Each column is the mean of four replicates and bars represent the standard error of the mean. Columns labeled with asterisk indicate significant difference at $P < 0.05$ between cultivars.
